# The Effect of Exercise Training on Diastolic and Systolic Function After Acute Myocardial Infarction: A Randomized Study

**DOI:** 10.1097/MD.0000000000001450

**Published:** 2015-09-11

**Authors:** Ricardo Fontes-Carvalho, Ana Isabel Azevedo, Francisco Sampaio, Madalena Teixeira, Nuno Bettencourt, Lilibeth Campos, Francisco Rocha Gonçalves, Vasco Gama Ribeiro, Ana Azevedo, Adelino Leite-Moreira

**Affiliations:** From the Cardiology Department, Gaia Hospital Centre, Gaia (RFC, AIA, FS, MT, NB, LC, VGR); Department of Physiology and Cardiothoracic Surgery (RFC, ALM); Department of Medicine (FRG); Department of Clinical Epidemiology, Predictive Medicine and Public Health, Faculty of Medicine, (AA); EPIUnit - Institute of Public Health, University of Porto (ISPUP) (AA); and Department of Cardiothoracic Surgery, Centro Hospitalar São João, Porto, Portugal (ALM).

## Abstract

After acute myocardial infarction (AMI), diastolic dysfunction is frequent and an important determinant of adverse outcome. However, few interventions have proven to be effective in improving diastolic function. We aimed to determine the effect of exercise training on diastolic and systolic function after AMI.

One month after AMI, 188 patients were prospectively randomized (1:1) to an 8-week supervised program of endurance and resistance exercise training (n = 86; 55.9 ± 10.8 years) versus standard of care (n = 89; 55.4 ± 10.3 years). All patients were submitted to detailed echocardiography and cardiopulmonary exercise test, at baseline and immediately after the study. Diastolic function was evaluated by the determination of tissue-Doppler derived early diastolic velocities (E′ velocity at the septal and lateral sides of mitral annulus) and by the E/E′ (ratio between the E wave velocity from mitral inflow and the E’ velocity) as recommended in the consensus document for diastolic function assessment.

At the end of the study, there was no significant change in E′ septal velocity or E/E′ septal ratio in the exercise group. We observed a small, although nonsignificant, improvement in E′ lateral (mean change 0.1 ± 2.0 cm/s; *P* = 0.40) and E/E′ lateral ratio (mean change of −0.3 ± 2.5; *P* = 0.24), while patients in the control group had a nonsignificant reduction in E′ lateral (mean change −0.4 ± 1.9 cm/s; *P* = 0.09) and an increase in E/E′ lateral ratio (mean change + 0.3 ± 3.3; *P* = 0.34). No relevant changes occurred in other diastolic parameters. The exercise-training program also did not improve systolic function (either tissue Doppler systolic velocities or ejection fraction).

Exercise capacity improved only in the exercise-training group, with an increase of 1.6 mL/kg/min in *p*VO_2_ (*P* = 0.001) and of 1.9 mL/kg/min in VO_2_ at anaerobic threshold (*P* < 0.001).

After AMI, an 8-week endurance plus resistance exercise-training program did not significantly improve diastolic or systolic function, although it was associated with an improvement in exercise capacity parameters.

## INTRODUCTION

Diastolic dysfunction is prevalent in the community^1^ and is recognized as an important predictor of heart failure^2^ and long-term mortality.^1,3^ After acute myocardial infarction (AMI), both systolic and diastolic functions are significantly impaired, and the majority of patients have diastolic dysfunction.^4–6^ In these patients, diastolic dysfunction is also a major determinant of adverse clinical outcome^4^ and decreased functional capacity.^5^ However, contrary to systolic function, no therapy or intervention has been shown to significantly improve diastolic function after AMI.^7^

Exercise training, as part of cardiac rehabilitation, is effective in improving functional capacity and quality of life in patients with coronary artery disease.^8,9^ Other cardiovascular and noncardiovascular benefits have been reported, namely in glucose metabolism, skeletal muscle function, oxidative stress, vascular function, pulmonary circulation, ischaemia-reperfusion lesion, and ventricular remodeling.^10^ However, the benefit of exercise training on diastolic function is controversial,^6,11,12^ especially after AMI where no longitudinal study has evaluated diastolic function using modern echocardiographic parameters.

We conducted a prospective, randomized, controlled study to determine the effect of exercise training on resting diastolic and systolic function in patients after acute myocardial infarction.

## METHODS

### Participants and Study Design

We prospectively enrolled patients 1 month after AMI, with both ST elevation and non-ST elevation AMI, defined according to the universal definition consensus document.^13^ Exclusion criteria were age below 18 or above 75 years, inability to exercise, hemodinamically significant valvular disease, moderate-to-severe chronic lung disease (vital capacity and/or forced expiratory volume in 1 s below 80% of age-dependent predicted value), atrial fibrillation, uncontrolled atrial or ventricular tachyarrhythmias, exercise-induced myocardial ischemia, severe renal disease or dysfunction (creatinine clearance <30 mL/min, calculated by the Cockcroft–Gault formula), and anaemia (haemoglobin <12 g/dL). Patients were randomized (1:1) to be included in a structured exercise-training program versus a control group, receiving standard of care. Randomization by blocks was used, and an allocation sequence based on a fixed block size of 8 was generated with a computer random number generator.

The study was conducted from January 2012 to January 2013, in the Cardiology Department of Gaia Hospital Center, which is a tertiary care hospital with a reference population of 700,000 patients. Immediately before enrolment and at the end of the study, all patients were submitted to clinical evaluation, detailed transthoracic echocardiography, and cardiopulmonary exercise test. Both groups had regular appointments with a cardiologist and received optimal medical therapy.

Informed consent was obtained from all patients and the local institution review board (“Comissão de Ética do Centro Hospitalar de Vila Nova de Gaia”) approved the study protocol (reference 627/10). The study protocol conforms to the principles outlined in the Declaration of Helsinki (1975) and the study has been registered at ClinicalTrials.gov (reference NCT02224495).

### Echocardiographic Evaluation

A single experienced cardiologist, blinded to the patient assignment group, performed all echocardiographic studies using an ultrasound system (iE33, Philips Medical Solutions, Best, The Netherlands) equipped with S5-1 and X5-1 transducer. Cardiac chamber dimensions, volumes, and left ventricular mass were measured according to current recommendations.^14^ Mitral inflow velocities were assessed using pulsed-wave Doppler in the apical four-chamber view, with the sample volume placed between the tips of the mitral leaflets; velocities were recorded at end-expiration. Tissue Doppler velocities were acquired at end-expiration, in the apical four-chamber view, with the sample positioned at the septal and lateral mitral annulus for determination of systolic (S′), early diastolic (E′), and late diastolic (A′) velocities. Pulsed wave Doppler velocities at the upper right pulmonary vein were also recorded. For all parameters, the average of 3 consecutive heart beats was recorded.

Left ventricle diastolic function was assessed according to the EAE/ASE consensus guidelines on diastolic function evaluation^15^ which included determination of peak early (E) and late (A) diastolic mitral inflow velocities, deceleration time of early left ventricular filling (DT), E/A ratio, myocardial early diastolic velocities at the septal and lateral side of mitral annulus (E′septal, E′lateral, E′mean), E/E′ ratio (including septal, lateral, and mean E/E′), pulmonary vein flow analysis (to calculate the Ard-Ad relation: the time difference between the duration of the atrial reverse wave of the pulmonary flow—Ard—and the mitral A-wave duration), and isovolumetric relaxation time (IVRT). Using the consensus criteria^15^ patients were categorized in diastolic dysfunction (DD) grades: normal, grade I (mild DD), grade II (moderate DD), and grade III (severe DD), by 2 blinded independent cardiologists. In case of discordance, each case was discussed individually.

Left ventricle systolic function was evaluated by calculation of biplane ejection fraction (Simpson's method) and determination of systolic velocities at the septal and lateral sides of mitral annulus by tissue Doppler (S'septal and S’lateral).

### Cardiopulmonary Exercise Testing

At the beginning and the end of the study, each patient underwent a symptom-limited cardiopulmonary exercise testing (CPX) on a treadmill, using the modified Bruce protocol (Cardiovit CS-200 Ergo Spiro; Schiller, Baar, Switzerland). Expired gases were continuously collected throughout exercise and analyzed for ventilatory volume (VE) and for oxygen (O_2_) and carbon dioxide (CO_2_) content, using dedicated analyzers. Standard spirometry (forced expiratory volume in 1 s (FEV1)) and forced vital capacity (FVC) were also undertaken before exercise test. Equipment calibration and all measurements were done according to the recommendations of the American Thoracic Society and American College of Chest Physicians.^16^ The following variables were calculated: peak oxygen consumption (pVO_2_) measured in milliliter per kilogram per minute (mL/kg/min); peak respiratory exchange ratio, defined by the ratio of CO_2_ production to O_2_ consumption at peak effort; anaerobic threshold (AT) defined as the point at which CO_2_ production increases disproportionately in relation to O_2_ consumption, obtained from a graph plotting O_2_ consumption against CO_2_ production; and total exercise duration (seconds). Patients were not asked to discontinue beta-blockers before the test.

### Intervention: The Exercise-Training Program

The exercise-training group participated in an 8-week outpatient exercise-training program, encompassing 3 sessions per week, including endurance and resistance training supervised by a physical therapist. Each session consisted of 10 minutes of warm-up, 50 minutes of aerobic and resistance training, and 10 minutes of cool-down. During the 8 weeks, endurance training (cycling on the first 4 weeks and treadmill on the remaining 4 weeks) of increasing intensity was performed. Training intensity was prescribed individually to each patient by an expert in cardiac rehabilitation, to a target heart rate of 70–85% of the maximal heart rate achieved in baseline CPX. Resistance training was also included in each session, including arms, legs, and thoracic exercises including dumbbell or weight training depending on the patient's condition and exercise capacity (usually starting with 2 kg hand weights, 10 repetitions, and progressively increasing). Clinical and training parameters were monitored including arterial blood pressure, heart rate (by telemetry or wrist device), glycaemia (in diabetic patients), and training level intensity. Borg scale was used to monitor fatigue. Patients randomized to the control group received standard of care, with regular appointments with the cardiologist, optimized medication, and recommendations on healthy lifestyle, which included smoking cessation, diet and weight control, and 30 min of moderate intensity aerobic exercise, at least 5 times per week.

The primary endpoint was the change in E′ lateral velocity. Secondary endpoints were other echocardiographic parameters of left ventricular diastolic (E′ septal velocity and E/E′ septal, lateral and mean ratio, E/A ratio) and systolic function (S’ septal and lateral velocities and ejection fraction) and parameters of exercise capacity (peak VO_2,_ VO_2_ at anaerobic threshold and exercise duration).

### Statistical Analysis

Statistical analysis was performed using SPSS Statistics 22.0 (IBM Corp, Armonk, NY). All continuous variables were expressed as mean ± standard deviation or as median (percentile 25–75) for variables with nonnormal distribution. Categorical variables were expressed as number (n) and percentage (%). A significance level of 0.05 was used. Differences between clinical and echocardiographic baseline characteristics of the 2 groups were assessed by an independent-samples t-test, *χ*^2^, or Mann–Whitney test, as appropriate. Changes in echocardiographic and cardiopulmonary test within groups were determined by paired-sample t-test (if normally distributed) or Wilcoxon test (if not normally distributed).

To calculate the sample size we used as reference the data from the study of Edelman et al, which tested the effect of exercise training.^17^ Assuming a standard deviation of the change in E′ velocity from baseline to follow-up of 1.5 cm/s in each group, we estimated that, to detect a difference of 1 cm/s in the change of E′ velocity between treatment groups, a minimum of 48 patients would be required in each group. A significance level of 5% and a statistical power of 90% were defined.

## RESULTS

**FIGURE 1:**
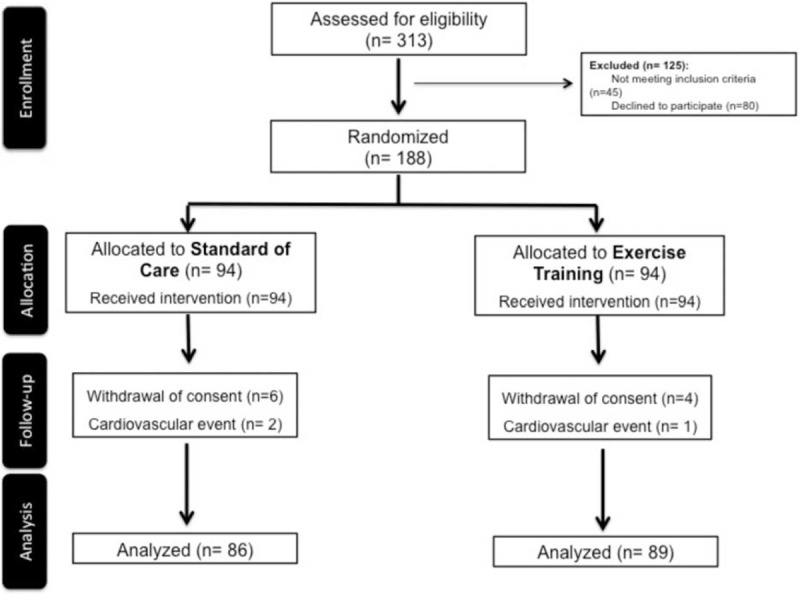
Flow chart of the study.

From 313 patients assessed for eligibility, 175 completed the protocol according to the study's flow chart outlined in Figure 1. The characterization of the study population is presented in Table 1. The study sample included mostly men (82.3%), with a mean age of 55.6 ± 10.5 years, an ejection fraction of 54.0 ± 9.5%, and an overall prevalence of DD of 61.1%: 26.9% had grade I DD; 29.3% had grade II, and 4.8% had grade III DD. Baseline demographic characteristics, cardiovascular risk factors, and echocardiographic parameters did not significantly differ between groups, as shown in Table 1. Regarding the main comorbidities, we excluded patients with chronic lung disease (moderate or severe), severe chronic renal disease and anaemia, but 12% had diabetes and 18.2% were obese. Patients received optimized medical therapy, with dual anti-platelet therapy in all patients, beta-blockers in 95.2%, renin-angiotensin axis inhibitor in 97.8%, aldosterone antagonist in 37.2%, and statins in 97.3%. There were no significant differences between the 2 groups in cardiovascular medical therapy. As shown in the flowchart (Figure 1), during the study, a total of 3 patients experienced a cardiovascular event (1 acute coronary syndrome in the coronary, 1 heart failure hospitalization, and 1 stroke), but none of these events occurred during or immediately after the exercise-training period. After a 2-year clinical follow-up, we further observed 3 major adverse cardiovascular events (MACE), which corresponded to 3 nonfatal myocardial infarctions in the group allocated to exercise training, and 1 MACE (1 cardiac death) in the group receiving standard of care.

**TABLE 1:**
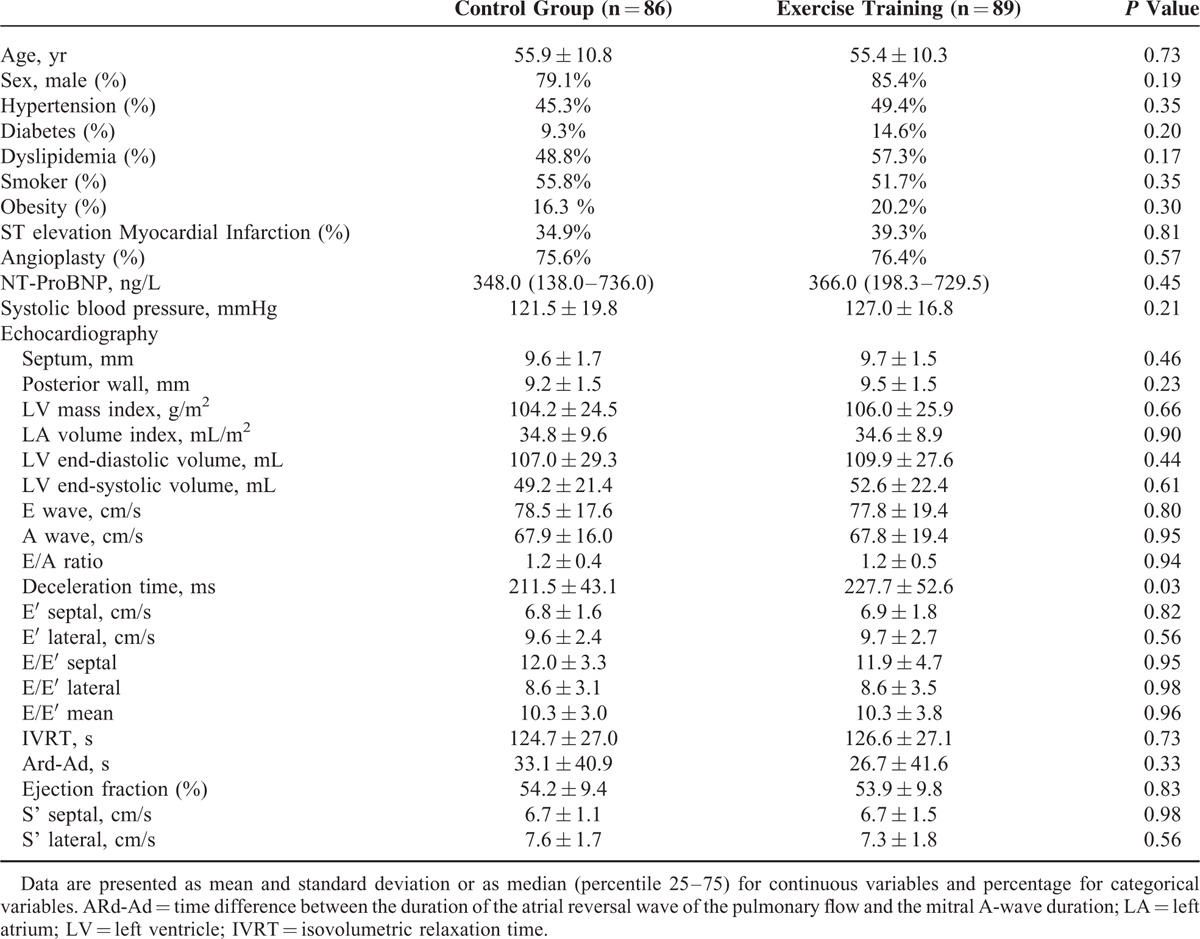
Baseline Characteristics of the Study Sample

### Effect of Exercise Training on Diastolic and Systolic Cardiac Function

As shown in Table 2, there was no significant improvement in E′ septal or E/E′ septal neither in the exercise training group nor in controls. At the end of the study, patients in the control group showed a nonsignificant reduction in E′ lateral (mean change of −0.4 ± 1.9 cm/s) and an increase in E/E′ lateral ratio (mean change of +0.3 ± 3.3) and in E/E′ mean ratio (change of + 0.2 ± 2.8), whereas in the exercise training group there was a slight, but also nonsignificant, improvement in E′ lateral (mean change of 0.1 ± 2.0 cm/s), E/E′ lateral ratio (mean change of −0.3 ± 2.5), and in E/E′ mean ratio (mean change −0.3 ± 2.8).

**TABLE 2:**
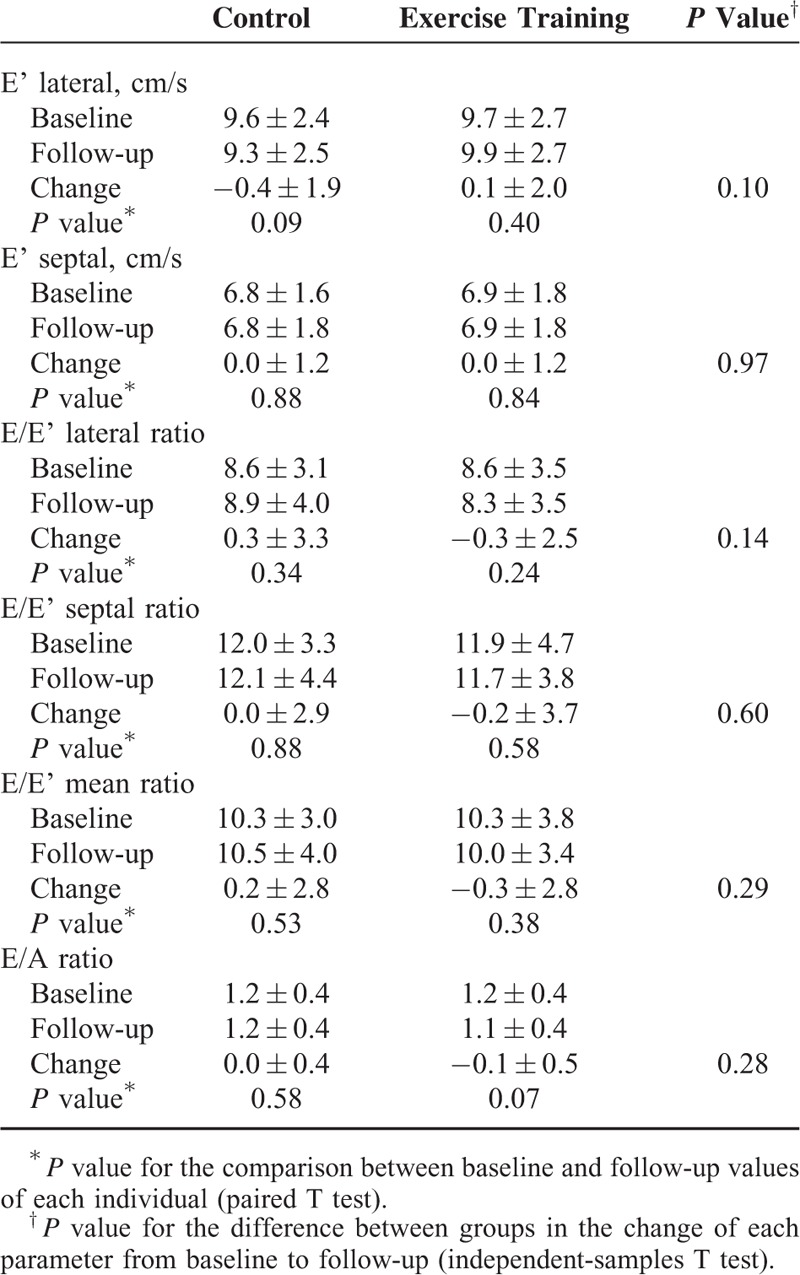
Comparison of Diastolic Function Parameters at Baseline and Follow-Up Between Control and Exercise Training Groups

Exercise training did not significantly improve other diastolic function parameters, such as E/A ratio, deceleration time, isovolumic relaxation time, or the Ard-Ad relation derived from pulmonary vein flow analysis. We did not observe a significant change in left atrium volume index in either group (34.7 ± 8.9 mL/m^2^ at baseline to 34.7 ± 10.9 mL/m^2^ at the end of the study, in the exercise-training group; *P* = 0.97).

Restricting the analysis only to patients with any grade of diastolic dysfunction, we did not observe a significant improvement in diastolic dysfunction parameters, namely in E′ lateral (*P* = 0.30), E′ septal (*P* = 0.15), or E/E′ mean ratio (*P* = 0.27).

There was no significant change in left ventricle ejection fraction in the 2 groups, as outlined in Table 3. Regarding the evaluation of systolic function by tissue Doppler parameters, we also did not observe any improvement in S’ septal or S’ lateral.

**TABLE 3:**
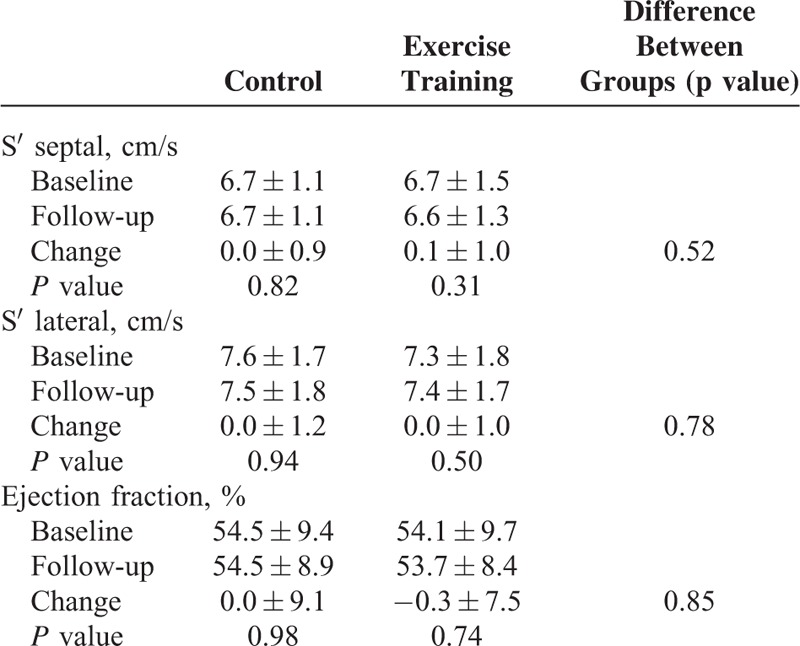
Comparison of Systolic Function Parameters at Baseline and Follow-Up Between Control and Exercise Training Groups

### Effect of Exercise Training on Functional Capacity

Exercise training significantly improved exercise capacity parameters, as shown in Table 4. At the end of the study, patients in the exercise-training group had an increase in peak VO_2_ (mean increase of + 1.9 ± 5.26 mL/min/kg; *P* < 0.01), in VO_2_ at anaerobic threshold (+1.4 ± 3.8 mL/min/kg; *P* < 0.01) and in exercise duration (+74.5 ± 76.9 s; *P* < 0.01), compared with baseline. Patients in the control group slightly improved exercise duration (+34.0 ± 73.6 s compared with baseline; *P* < 0.01) but there was no increase in peak VO_2_ or in VO_2_ at anaerobic threshold.

**TABLE 4:**
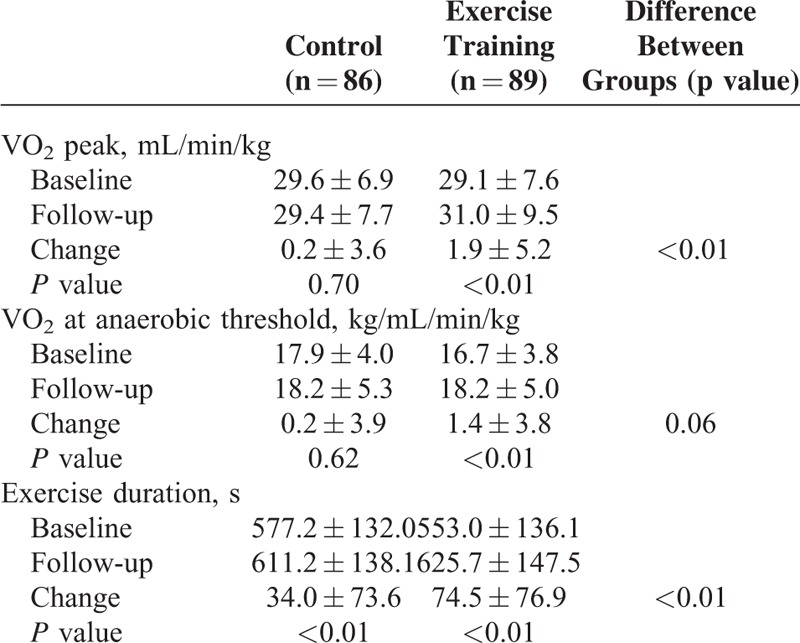
Comparison of Capacity Parameters at Baseline and Follow-Up Between Control and Exercise Training Groups

## DISCUSSION

In this prospective, randomized, controlled study an 8-week exercise-training program after myocardial infarction did not significantly improve diastolic or systolic function parameters, although it was associated with a significant improvement in exercise capacity.

In patients after myocardial infarction, left ventricle diastolic dysfunction is frequent^4,5,6^ and is associated with an adverse clinical outcome.^4^ In our study, using the most recent consensus definition for diastolic function evaluation,^15^ we confirmed a high prevalence (61.1%) of diastolic dysfunction in this population. It is also known that diastolic dysfunction is also an important determinant of exercise intolerance,^5^ but no interventions have proven to significantly improve diastolic function.^7^

### Impact of Exercise on Left Ventricular Diastolic Function

Data from animal studies suggested that endurance training could improve myocardial relaxation and calcium homeostasis, by increasing the myocardial expression of SERCA2a and phospholamban.^18,19^ In patients with systolic heart failure, the benefit of exercise on diastolic function is controversial,^11,20^ but in patients with heart failure with preserved ejection fraction, recent studies have shown that exercise can improve diastolic function parameters.^17^ However, after AMI few longitudinal studies have evaluated the impact of exercise training on left ventricular diastolic function^6^ and none of them has used modern and integrated echocardiographic parameters for diastolic function assessment. In our study, we observed that this 8-week structured exercise-training program, consisting of endurance plus resistance training, did not significantly improve diastolic function because there was no significant change in lateral or septal E′ velocities and no reduction in left ventricle filling pressures estimated by E/E′ ratio.

The potential benefits of exercise training on myocardial structure and function can be influenced by several factors, such as the underlying cardiovascular disease, the baseline systolic and diastolic function, the criteria used for diastolic function evaluation, and the timing, type (endurance versus resistance), and duration of exercise. In this study, we tested a combination of endurance plus resistance training during a relatively short period of time. First, it is possible that an 8-week duration exercise program was too short to induce changes in myocardial function. However, in the study from Edelmann et al,^17^ including patients with heart failure preserved ejection fraction, there was a significant improvement in E/E′ ratio already at 12 weeks. On the contrary, another study consisting of 16-week exercise training (combining endurance plus strength training in the last 8 weeks) failed to improve cardiac function in patients with diastolic dysfunction.^11^ Second, the type of exercise could be responsible for these results.^21^ The present study incorporated traditional endurance training, which was complemented with a strength-training component and was very similar to the training protocol used by Edelman et al.^17^ In this setting, strength training can be useful to accelerate improvements in skeletal muscle bulk and function. Newer training modalities using high-intensity aerobic interval training (reaching 95% of peak heart rate) seem to be superior to moderate continuous endurance training, for improving ejection fraction, endothelial function, and skeletal muscle function in patients with systolic heart failure.^22^ Third, the timing of initiation of exercise can also influence the effect of exercise training. Although the guidelines do not provide specific recommendations, most cardiac rehabilitation programs commence at least 4–6 weeks after hospital discharge.^23^ However, previous studies have suggested that the largest improvements in ventricular volumes and ejection fraction can be obtained when exercise is began just 1 week after AMI.^24^ This latter study did not evaluate the effect on diastolic function.

In this study, diastolic function was evaluated according to the latest recommendations from the consensus document between the European Association of Echocardiography and the American Society of Echocardiography^15^ which recommend the evaluation of E′ velocities and E/E′ ratio from tissue Doppler. It is known that the E′ velocity is a preload-independent index of LV relaxation,^25^ whereas the E/E′ ratio can be used to estimate increased left ventricle filling pressures.^26,27^ They have been shown to be important prognostic markers under several cardiac conditions.^28^ Nevertheless, in specific clinical scenarios, the evaluation of diastolic function by tissue Doppler can be challenging,^29^ such as in patients with decompensated systolic heart failure,^30^ cardiac resynchronization therapy, or healthy individuals.^31^ More recently, new techniques for the evaluation of ventricular and atrium function have emerged such as strain and strain rate. Their role in the evaluation of diastolic function is not yet established and recent comparative invasive studies suggested that strain rate is not superior to tissue Doppler analysis such as the E/E′ ratio.^32^

### Effect of Exercise Training on Left Ventricular Systolic Function

It is also controversial if resting left ventricle systolic function can be improved by exercise training.^6,33,34^ Most of the studies were small, lacked control groups, and evaluated systolic function only using ejection fraction. It is known that ejection fraction is preload and afterload dependent and has several limitations in the assessment of global left ventricle systolic function. In this study, we evaluated systolic function also using tissue Doppler-derived systolic mitral annulus velocities (S’ lateral and septal velocities) that is known to be more sensitive in the evaluation of global and regional systolic function after myocardial infarction.^35^ However, in our study, neither ejection fraction nor S′ velocities significantly improved after the implementation of this exercise training program.

### Effect of Exercise Training on Functional Capacity

Contrary to cardiac function, the exercise training protocol significantly improved functional capacity, as determined by a peak VO_2_. These observations suggest that the improvement in functional capacity was caused mainly by noncardiac mechanisms, similar to what has been observed in heart failure patients.^22,36^ These mechanisms may include improved oxidative capacity or anaerobic glycolysis of skeletal muscle, improved arteriovenous difference (with better oxygen uptake by the peripheral tissues), and/or improved vascular function.^11,22^ In another study, Smart et al^10^ also showed that in patients with diastolic dysfunction the improvement in functional capacity parameters after 16 weeks of training was related to improvements in noncardiac parameters.

### Strengths and Limitations

This was a prospective, randomized, single-blinded, controlled trial that evaluated the effect of a supervised exercise-training program on diastolic and systolic function. For the assessment of cardiac function, we used recent echocardiographic parameters derived from tissue Doppler analysis, which provide a more accurate evaluation of left ventricular function in comparison with classical parameters. Also, we compared exercise capacity before and after the program by evaluating peak VO_2_ that is considered the gold-standard method to evaluate exercise tolerance.

Regarding the limitations of this study we investigated mostly middle-aged men and, therefore, no assumptions can be made regarding older individuals or women. Due to the study design, we evaluated the endpoints using a per protocol analysis. Cardiac function was evaluated only at rest, and therefore potential benefits of exercise training on cardiac function during exercise could not be evaluated. Moreover, we cannot exclude that patients had subclinical changes in cardiac function (especially in diastolic function) before the acute event. We tested the effect of an exercise-training protocol that was of relatively short duration and consisting of both endurance plus strength training protocol. However, in patients after AMI the optimal type, duration, frequency, and intensity of exercise training still needs to be addressed in the future. Finally, the contribution of noncardiac factors (peripheral and muscular) to the improvement of exercise capacity should be addressed in forthcoming studies.

## CONCLUSION

In patients 1 month after acute myocardial infarction, an 8-week structured exercise-training program, consisting of endurance plus resistance training, improved exercise capacity, but was not associated with a significant change in tissue Doppler parameters of diastolic and systolic left ventricular function.

Future studies will need to evaluate the effect of long-term exercise-training protocols on cardiac function, especially on diastolic function.
